# Supplementary material for: Do questionnaires reflect their purported cognitive functions?
Source: Cognition. 2020 Feb;195:104114. doi: 10.1016/j.cognition.2019.104114 (PMC6963768; doi:10.1016/j.cognition.2019.104114)
Supplement: Supplementary file 1 [file mmc1.docx]

**Supplementary Materials**

**Methods**

**Scene construction test** ([Hassabis, Kumaran, Vann, & Maguire, 2007](#_ENREF_1)). This test measures a participant’s ability to mentally construct a visual scene. Participants construct seven different scenes of commonplace settings. For each scene, a short cue is provided (e.g. imagine lying on a beach in a beautiful tropical bay), and the participant is asked to imagine the scene that is evoked and then describe it out loud in as much detail as possible. Participants are explicitly told not to describe a memory, but to create a new scene that they have never experienced before. Participants give descriptions until they come to a natural end or cannot add any additional details. If required, a probing protocol is utilised to attempt to elicit more details (if a description is particularly poor); these are either very general probes (e.g. is there anything else you can tell me?) or based upon a theme described by the participant. The experimenter is never allowed to introduce new concepts or details that have not been mentioned by the participant. All descriptions are audio recorded and transcribed for scoring.

Following each scene description, participants rate their imagined scene in terms of their feeling of sense of presence (1 - ‘did not feel like I was there at all’…5 - ‘felt strongly like I was really there’) and perceived vividness (1 - ‘couldn’t really see anything’…5 - ‘extremely vivid’). They also rate the spatial coherence of the scene, choosing from twelve statements providing possible qualitative descriptions of the scene. Participants are instructed to indicate the statements they feel accurately described their construction. Eight of these statements describe a spatially coherent scene (e.g. ‘I could see the whole scene in my mind’s eye’) and 4 describe a fragmented scene (e.g. ‘It was a collection of separate images’). Participants can choose as many or as few options as they feel were relevant to the scene.

The overall outcome measure from the scene construction test is an “experiential index” which is calculated for each scene and then averaged. It is composed of four elements: numerical scoring of the content, participant ratings of their sense of presence (how much they felt like they were really there) and perceived vividness, participant ratings of the spatial coherence of the scene, and an experimenter rating of overall quality of the scene. To obtain the content score, the transcribed versions of each scene are split into statements. These are then classified by the experimenter as belonging to one of four categories: spatial references, entities present, sensory descriptions, or thoughts/emotions/actions. A maximum of seven details per category can be awarded (providing a maximum content score of 28). The original study reporting this test determined that seven details per category is enough to create a coherent scene without over-rewarding more verbose participants ([Hassabis et al., 2007](#_ENREF_1)).

The participant ratings are the scores provided for sense of presence and perceived vividness, rescaled to 0-4, providing a maximum total of 8. Spatial coherence is from the qualitative statements that participants chose following each scene. One point is awarded for each coherent statement selected and one point taken away for each fragmented statement. This yields a score between –4 and +8 that is then normalised around zero to give a final spatial coherence index score ranging between –6 and +6. Only positive spatial coherence scores are included in the experiential index so as not to over penalise fragmented descriptions.

Finally, the experimenter assesses the overall quality of the scene on a scale between 0-10 (0 indicating a scene with no details and 10 a rich and vivid description). This was rescored to provide a rating from 0 to 18. The final experiential index score therefore ranged from 0 to 60; 28 points from the content, 8 for the participant ratings, 6 for the spatial coherence statements and 18 for the quality rating. An overall experiential index score was calculated by averaging across the 7 scenes.

Double scoring was performed on the data from 20% of the participants (i.e. 44 participants, which equates to 308 individual scenes). Scenes were scored by four experimenters, with double scoring performed on 20% of each experimenter’s scoring. We took the most stringent approach to identifying across-experimenter agreement. An inter-class correlation coefficient, with a two-way random effects model looking for absolute agreement, was calculated for each content score and for the quality ratings. This was performed both for individual scenes and as an average of all seven scenes across each participant. All inter-class correlation coefficients were above 0.9 (full details in Table S1). For reference, a score of 0.8 or above is considered excellent agreement beyond chance.

**Table S1.** Double scoring of the scene construction test.

|  | | **Rating** | | | | |
| --- | --- | --- | --- | --- | --- | --- |
|  | **Spatial References** | | **Entities present** | **Sensory Descriptions** | **Thoughts/**  **Emotions/Actions** | **Quality Ratings** |
| **For each individual scene** | | | | | | |
|  |  | |  |  |  |  |
| n = 308 | 0.90 | | 0.96 | 0.94 | 0.90 | 0.90 |
|  |  | |  |  |  |  |
| **For each individual participant (i.e. the score is averaged across the seven scenes)** | | | | | | |
|  | | | | | | |
| n = 44 | 0.91 | | 0.99 | 0.97 | 0.91 | 0.93 |
|  |  | |  |  |  |  |

**Autobiographical Interview** ([AI; Levine, Svoboda, Hay, Winocur, & Moscovitch, 2002](#_ENREF_2)). The AI asks participants to recall and describe autobiographical memories from a specific time and place over four time periods – early childhood (up to age 11), teenage years (from 11-17 years of age), adulthood (from aged 18 up to 12 months prior to the interview; two memories were requested) and the last year (a memory from the last 12 months). Participants are asked to avoid selecting a memory from the last 12 months in the adulthood category to ensure that only the last year category contained memories from the last 12 months. If a participant cannot spontaneously produce a memory, a cue card containing ~100 typical life events is presented to aid recall.

Participants are asked to select events that they are comfortable to talk about. They are told that the event has to be one where they were personally involved and of which they have a clear recollection (they could not just have been told about the event by others). All memories have to be from a specific time and place – an event for example could not be a two week summer holiday, but a specific event on that holiday would be acceptable. Participants are first asked to simply describe and speak about the event selected. This occurs without interruption from the experimenter until they have reached a natural end point. On completion, the experimenter prompts the participant with a general probe (e.g. is there anything more you can tell me?) to ascertain if any additional details can be elicited. All memories are audio recorded and transcribed for analysis.

For scoring, each memory is divided into segments of information. Segments are defined as a specific occurrence, observation or thought. Segments are then defined as either “internal” or “external”. Internal details are those describing the event in question. Internal details are split into five sub-categories: events (a specific detail of the story), places, time, perceptual information and emotions/thoughts. The number of details from each sub-category is summed to create the overall “internal details” measure. External details describe semantic information concerning the event, or non-event information. The number of internal and external details was combined to provide a total number of utterances, allowing for the number of internal details to be represented as a percentage of the total number of utterances to control for verbosity.

Double scoring was performed on the data from 20% of the participants (i.e. 44 participants, which equates to 215 individual memories). Memories were scored by three experimenters, with double scoring performed on 20% of each experimenter’s scoring. An inter-class correlation coefficient, with a two-way random effects model looking for absolute agreement was calculated for each internal detail sub-category, and the overall internal and external details. This was performed both for individual memories and as an average of all five memories across each participant. The majority of inter-class correlation coefficients were above 0.9, with the lowest at 0.81 (full details in Table S2).

**Table S2.** Double scoring of the Autobiographical Interview.

|  | | **Rating** | | | | | | |
| --- | --- | --- | --- | --- | --- | --- | --- | --- |
|  | **Internal Event** | | **Internal Place** | **Internal Time** | **Internal Perceptual** | **Internal Emotion** | **Internal Details** | **External Details** |
| **For each individual memory** | | | | | | |  |  |
|  |  | |  |  |  |  |  |  |
| n = 215 | .92 | | .85 | .94 | .92 | .86 | **.94** | **.84** |
|  |  | |  |  |  |  |  |  |
| **For each individual participant (i.e. score is averaged across the five memories)** | | | | | | | | |
|  | | | | | | |  |  |
| n = 43 | .95 | | .88 | .96 | .94 | .81 | **.97** | **.87** |
|  |  | |  |  |  |  |  |  |

**Future thinking test** ([Hassabis et al., 2007](#_ENREF_1)). Double scoring was performed on the data from 20% of the participants (i.e. 44 participants, which equates to 132 future scenes). Data were scored by four experimenters, with double scoring performed on 20% of each experimenter’s scoring – see Table S3.

**Table S3.** Double scoring of the future thinking test.

|  | | **Rating** | | | | |
| --- | --- | --- | --- | --- | --- | --- |
|  | **Spatial References** | | **Entities present** | **Sensory Descriptions** | **Thoughts/**  **Emotions/Actions** | **Quality ratings** |
| **For each individual scene** | | | | | | |
|  |  | |  |  |  |  |
| n = 132 | 0.90 | | 0.94 | 0.93 | 0.88 | 0.90 |
|  |  | |  |  |  |  |
| **For each individual participant (i.e. the score is averaged across the three future events)** | | | | | | |
|  | | | | | | |
| n = 44 | 0.94 | | 0.95 | 0.96 | 0.88 | 0.92 |
|  |  | |  |  |  |  |

**Navigation tests** ([Woollett & Maguire, 2010](#_ENREF_3)). Navigation ability was assessed using movies of navigation through an unfamiliar town. Movie clips of two overlapping routes through this real town (Blackrock, in Dublin, Ireland) are shown to the participant four times. Footage is unique to each route apart from one crossover point at a large road junction. The footage was shot at eye level and proceeds at an average walking pace, with the camera panning from side to side to simulate viewing and to allow for observation of the features and landmarks along the routes. At road junctions, the pace is slowed to allow for all elements of the junction to be observed before continuing. The movies are shown without sound. Participants are told to focus on salient landmarks to help them learn the town, ignoring cars, buses and people. Landmarks are defined as prominent buildings and distinctive elements of the route. Participants are explicitly told that the two routes, while shown separately, overlapped.

Five tasks are used to assess the participant’s ability to learn the new town, with an overall navigation score calculated by combining the scores from all the tasks. First, following each viewing of the route movies, participants are shown four short clips – two from the actual routes, and two distractors. Participants indicate whether they had seen that clip or not. The final score (/16) is the number of correctly identified clips.

Second, after all four route viewings are completed, recognition memory for scenes from the routes is tested. Participants are shown 32 photographs, 24 from the routes (12 from each route) and 8 similar distractors, randomly intermixed. Participants have to report whether they had seen that scene or not. The final score (/32) is the number of correctly identified scenes.

A third test involves assessing knowledge of the spatial relationships between landmarks from the routes. On each trial, three colour photographs of landmarks are presented and participants have to judge which of two of the landmarks was closer, as the crow flies, to the third picture (i.e. the target landmark). Ten trials are conducted, 6 where the landmarks are all from the same route (3 from each route) and 4 where the landmarks are from across the two routes. The final score is the number of correct judgements (/10).

Fourth, route knowledge is examined by having participants place photographs from the routes in the correct order as if travelling through the town. On each trial, participants are given eight photographs, one marked as the “start point” and another as the “end point”. They are then asked to place the other six photographs in the correct order to get from the start to the end. Four trials are performed, two that remain within one route and two that involve both routes. Correctly placed photographs are given a score of 1 (the maximum being 24).

Finally, participants draw a sketch map of the two routes including as many landmarks as they can remember (with it being made clear that drawing ability was not being assessed). Sketch maps are scored in terms of:

- The number of road segments (a segment being the section of road between road junctions, out of 16)
- The number of road junctions (out of 8)
- The number of correct landmarks (out of 34 identifiable landmarks)
- Landmark placement (up to 3 points per landmark; 1 for the correct side of the road, 1 for placement with regards to nearby road junctions and 1 for being in the correct sequence of nearby landmarks. Total maximum of 102)
- The orientation of the routes (an experimenter score assessing the orientation and layout of the map from 1-5. 1 being poor representation of the town, 5 being accurate orientation)
- An overall map categorisation score (an experimenter score representing map coherence from 1-6; 1: The two routes are merged. 2: Two routes are present, but drawn separately. 3: Routes are close together but not joined accurately. 4: Some elements of the routes are linked, but integration is mainly lacking. 5: The two routes are integrated, but with some inaccuracies. 6: Correct integration, easy to follow and use for navigating.

Sketch maps were scored by three experimenters, with double scoring performed on 20% of each experimenter’s scoring (n = 42 maps). All the inter-class correlation coefficients were above 0.89 (Table S4).

**Table S4.** Double scoring of the navigation sketch maps.

|  | **Rating** | | | | | | |
| --- | --- | --- | --- | --- | --- | --- | --- |
|  | | **Road Segments** | **Road Junctions** | **Number of Landmarks** | **Landmark Placement** | **Map Orientation** | **Map Categorisation** |
|  | |  |  |  |  |  |  |
| n = 42 | | 0.95 | 0.96 | 0.97 | 0.96 | 0.96 | 0.89 |
|  | |  |  |  |  |  |  |

**Table S5.** Correlation coefficients of the memory questionnaires with AI internal details as a percentage of total utterances for each memory time point.

|  | **Childhood** | | **Teenage** | | **“Remote”** | | **Adult** | | **Last Year** | | **“Recent”** | |
| --- | --- | --- | --- | --- | --- | --- | --- | --- | --- | --- | --- | --- |
|  | **r** | **p** | **r** | **p** | **r** | **p** | **r** | **p** | **r** | **p** | **r** | **p** |
|  |  |  |  |  |  |  |  |  |  |  |  |  |
| Memory Experience Questionnaire; Accessibility | 0.015 | 0.82 | -0.053 | 0.44 | -0.023 | 0.74 | 0.13 | 0.048 | 0.063 | 0.36 | 0.12 | 0.074 |
| Memory Experience Questionnaire; Coherence | -0.030 | 0.66 | -0.21 | 0.002 | -0.14 | 0.039 | -0.14 | 0.042 | -0.094 | 0.17 | -0.14 | 0.043 |
| Memory Experience Questionnaire; Sharing | -0.017 | 0.80 | -0.048 | 0.48 | -0.039 | 0.57 | -0.004 | 0.95 | 0.12 | 0.071 | 0.048 | 0.48 |
| Memory Experience Questionnaire; Vividness | -0.002 | 0.98 | -0.083 | 0.23 | -0.051 | 0.46 | 0.005 | 0.94 | 0.002 | 0.98 | 0.004 | 0.95 |
| Subjective Memory Questionnaire | 0.054 | 0.43 | -0.080 | 0.24 | -0.017 | 0.80 | 0.005 | 0.95 | -0.001 | 0.99 | 0.003 | 0.97 |
| Survey of Autobiographical Memory; Episodic | -0.064 | 0.35 | -0.14 | 0.036 | -0.12 | 0.071 | -0.053 | 0.44 | -0.007 | 0.92 | -0.040 | 0.55 |
|  |  |  |  |  |  |  |  |  |  |  |  |  |

**Table S6.** Correlation coefficients of the other group questionnaires with AI internal details as a percentage of total utterances for each memory time point.

|  | **Childhood** | | **Teenage** | | **“Remote”** | | **Adult** | | **Last Year** | | **“Recent”** | |
| --- | --- | --- | --- | --- | --- | --- | --- | --- | --- | --- | --- | --- |
|  | **r** | **p** | **r** | **p** | **r** | **p** | **r** | **p** | **r** | **p** | **r** | **p** |
| **Imagery Questionnaires** |  |  |  |  |  |  |  |  |  |  |  |  |
|  |  |  |  |  |  |  |  |  |  |  |  |  |
| OSIVQ; Object-Scene | -0.11 | 0.11 | -0.081 | 0.24 | -0.11 | 0.10 | -0.097 | 0.16 | -0.060 | 0.38 | -0.094 | 0.17 |
| OSIVQ; Spatial | -0.015 | 0.82 | -0.038 | 0.58 | -0.032 | 0.64 | -0.093 | 0.17 | -0.11 | 0.11 | -0.11 | 0.10 |
| PSIQ; Appearance | -0.055 | 0.42 | -0.013 | 0.85 | -0.040 | 0.56 | -0.022 | 0.75 | -0.061 | 0.37 | -0.041 | 0.55 |
| Spontaneous Use of Imagery Scale | -0.12 | 0.092 | 0.017 | 0.80 | -0.056 | 0.41 | -0.13 | 0.064 | -0.042 | 0.53 | -0.11 | 0.12 |
| Visualizer | -0.075 | 0.27 | -0.076 | 0.26 | -0.089 | 0.19 | -0.072 | 0.29 | -0.099 | 0.15 | -0.092 | 0.18 |
|  |  |  |  |  |  |  |  |  |  |  |  |  |
| **Future Thinking Questionnaire** |  |  |  |  |  |  |  |  |  |  |  |  |
|  |  |  |  |  |  |  |  |  |  |  |  |  |
| Survey of Autobiographical Memory; Future | -0.030 | 0.66 | -0.073 | 0.28 | -0.061 | 0.37 | -0.056 | 0.41 | -0.040 | 0.56 | -0.056 | 0.41 |
|  |  |  |  |  |  |  |  |  |  |  |  |  |
| **Navigation Questionnaires** |  |  |  |  |  |  |  |  |  |  |  |  |
|  |  |  |  |  |  |  |  |  |  |  |  |  |
| Santa Barbara Sense of Direction Scale | -0.009 | 0.89 | -0.045 | 0.51 | -0.033 | 0.63 | -0.084 | 0.22 | -0.067 | 0.32 | -0.087 | 0.20 |
| Survey of Autobiographical Memory; Spatial | -0.041 | 0.55 | -0.075 | 0.27 | -0.069 | 0.31 | -0.075 | 0.27 | -0.13 | 0.066 | -0.11 | 0.12 |
|  |  |  |  |  |  |  |  |  |  |  |  |  |
| **Verbal and Semantic Memory Questionnaires** |  |  |  |  |  |  |  |  |  |  |  |  |
|  |  |  |  |  |  |  |  |  |  |  |  |  |
| OSIVQ; Verbal | 0.065 | 0.34 | -0.058 | 0.39 | 0.003 | 0.97 | -0.098 | 0.15 | 0.054 | 0.43 | -0.047 | 0.49 |
| Survey of Autobiographical Memory; Semantic | 0.14 | 0.037 | 0.016 | 0.82 | 0.092 | 0.18 | 0.14 | 0.047 | 0.056 | 0.41 | 0.12 | 0.079 |
| Verbalizer | -0.056 | 0.41 | -0.15 | 0.024 | -0.13 | 0.067 | -0.16 | 0.016 | -0.057 | 0.40 | -0.14 | 0.039 |
|  |  |  |  |  |  |  |  |  |  |  |  |  |

**Table S7.** Correlation coefficients of the One Sentence Questionnaire questions with AI internal details as a percentage of total utterances for each memory time point.

|  | **Childhood** | | **Teenage** | | **“Remote”** | | **Adult** | | **Last Year** | | **“Recent”** | |
| --- | --- | --- | --- | --- | --- | --- | --- | --- | --- | --- | --- | --- |
|  | **r** | **p** | **r** | **p** | **r** | **p** | **r** | **p** | **r** | **p** | **r** | **p** |
| **Imagery Questions** |  |  |  |  |  |  |  |  |  |  |  |  |
| One Sentence: Imagery Ability | -0.054 | 0.43 | -0.038 | 0.58 | -0.054 | 0.43 | -0.039 | 0.57 | -0.039 | 0.57 | -0.044 | 0.52 |
| One Sentence: Imagery Use | -0.070 | 0.31 | -0.072 | 0.29 | -0.084 | 0.22 | -0.045 | 0.51 | -0.092 | 0.18 | -0.070 | 0.30 |
| One Sentence: Imagery as a Scene | -0.032 | 0.64 | -0.081 | 0.23 | -0.068 | 0.32 | -0.027 | 0.69 | -0.13 | 0.066 | -0.071 | 0.30 |
|  |  |  |  |  |  |  |  |  |  |  |  |  |
|  |  |  |  |  |  |  |  |  |  |  |  |  |
| **Memory Questions** |  |  |  |  |  |  |  |  |  |  |  |  |
| One Sentence: Memory Ability | 0.004 | 0.96 | -0.074 | 0.28 | -0.042 | 0.54 | 0.003 | 0.96 | -0.033 | 0.63 | -0.012 | 0.87 |
| One Sentence: Memory in Imagery | 0.016 | 0.82 | -0.020 | 0.77 | -0.003 | 0.97 | 0.052 | 0.44 | -0.012 | 0.86 | 0.032 | 0.64 |
| One Sentence: Memory in Scene Imagery | -0.018 | 0.79 | 0.027 | 0.69 | 0.005 | 0.94 | 0.090 | 0.19 | -0.023 | 0.73 | 0.054 | 0.43 |
| One Sentence: Memory in Words | 0.008 | 0.91 | -0.043 | 0.53 | -0.022 | 0.75 | -0.12 | 0.092 | 0.017 | 0.81 | -0.075 | 0.27 |
|  |  |  |  |  |  |  |  |  |  |  |  |  |
| **Future Thinking Questions** |  |  |  |  |  |  |  |  |  |  |  |  |
| Future Thinking Ability | -0.049 | 0.47 | -0.072 | 0.29 | -0.072 | 0.29 | -0.076 | 0.26 | 0.00 | 1.0 | -0.054 | 0.43 |
| Future Thinking in Imagery | 0.018 | 0.79 | -0.027 | 0.69 | -0.006 | 0.93 | 0.016 | 0.81 | -0.024 | 0.73 | 0.002 | 0.98 |
| Future Thinking in Scene Imagery | -0.047 | 0.49 | -0.067 | 0.33 | -0.067 | 0.33 | -0.024 | 0.73 | -0.030 | 0.66 | -0.029 | 0.67 |
| Future Thinking in Words | -0.033 | 0.63 | -0.15 | 0.027 | -0.11 | 0.11 | -0.18 | 0.008 | -0.051 | 0.46 | -0.15 | 0.028 |
|  |  |  |  |  |  |  |  |  |  |  |  |  |
| **Navigation Questions** |  |  |  |  |  |  |  |  |  |  |  |  |
| One Sentence: Navigation Ability | -0.012 | 0.87 | -0.037 | 0.59 | -0.029 | 0.67 | -0.12 | 0.080 | -0.10 | 0.14 | -0.13 | 0.063 |
| One Sentence: Navigation in Imagery | 0.071 | 0.30 | -0.018 | 0.80 | 0.031 | 0.65 | -0.083 | 0.22 | -0.048 | 0.48 | -0.079 | 0.25 |
| One Sentence: Navigation in Scene Imagery | -0.019 | 0.78 | -0.029 | 0.67 | -0.028 | 0.68 | -0.11 | 0.099 | -0.057 | 0.41 | -0.10 | 0.13 |
| One Sentence: Navigation in Words | -0.045 | 0.51 | -0.013 | 0.85 | -0.034 | 0.62 | 0.013 | 0.85 | 0.022 | 0.75 | 0.019 | 0.79 |
|  |  |  |  | |  | |  | |  | |  | |

**Table S8.** Correlation coefficients of the memory questionnaires with the AI internal detail sub-categories as a percentage of total utterances.

|  | **AI Events** | | **AI Place** | | **AI Time** | | **AI Perceptual** | | **AI Emotion** | |
| --- | --- | --- | --- | --- | --- | --- | --- | --- | --- | --- |
|  | **r** | **p** | **r** | **p** | **r** | **p** | **r** | **p** | **r** | **p** |
|  |  |  |  |  |  |  |  |  |  |  |
| Memory Experience Questionnaire; Accessibility | 0.071 | 0.30 | -0.12 | 0.085 | 0.098 | 0.15 | 0.013 | 0.85 | 0.023 | 0.74 |
| Memory Experience Questionnaire; Coherence | -0.074 | 0.28 | -0.12 | 0.077 | 0.12 | 0.068 | -0.009 | 0.90 | -0.12 | 0.076 |
| Memory Experience Questionnaire; Sharing | 0.079 | 0.25 | -0.19 | 0.006 | 0.015 | 0.83 | 0.009 | 0.89 | 0.021 | 0.75 |
| Memory Experience Questionnaire; Vividness | 0.025 | 0.71 | -0.16 | 0.017 | 0.073 | 0.29 | -0.044 | 0.52 | 0.056 | 0.41 |
| Subjective Memory Questionnaire | -0.051 | 0.46 | -0.053 | 0.44 | 0.099 | 0.15 | 0.055 | 0.42 | -0.038 | 0.58 |
| Survey of Autobiographical Memory; Episodic | -0.004 | 0.95 | -0.16 | 0.021 | 0.11 | 0.11 | -0.049 | 0.47 | -0.023 | 0.74 |
|  |  |  |  |  |  |  |  |  |  |  |

**Table S9**. Correlation coefficients of the other group questionnaires with the AI internal detail sub-categories as a percentage of total utterances.

|  | **AI Events** | | **AI Place** | | | | **AI Time** | | | | **AI Perceptual** | | | | **AI Emotion** | | | |  |
| --- | --- | --- | --- | --- | --- | --- | --- | --- | --- | --- | --- | --- | --- | --- | --- | --- | --- | --- | --- |
|  | **r** | **p** | | **r** | | **p** | | **r** | | **p** | | **r** | | **p** | | **r** | | **p** | |
| **Imagery Questionnaires** |  |  | |  | |  | |  | |  | |  | |  | |  | |  | |
|  |  |  | |  | |  | |  | |  | |  | |  | |  | |  | |
| OSIVQ; Object-Scene | -0.015 | 0.83 | | -0.15 | | 0.024 | | 0.078 | | 0.25 | | -0.084 | | 0.22 | | -0.003 | | 0.97 | |
| OSIVQ; Spatial | -0.035 | 0.60 | | -0.027 | | 0.70 | | -0.004 | | 0.95 | | -0.12 | | 0.070 | | 0.084 | | 0.22 | |
| PSIQ; Appearance | 0.014 | 0.84 | | -0.16 | | 0.016 | | 0.11 | | 0.11 | | -0.012 | | 0.86 | | -0.050 | | 0.47 | |
| Spontaneous Use of Imagery Scale | -0.022 | 0.75 | | -0.13 | | 0.054 | | 0.006 | | 0.93 | | -0.074 | | 0.28 | | 0.043 | | 0.53 | |
| Visualizer | -0.074 | 0.28 | | -0.015 | | 0.83 | | -0.033 | | 0.63 | | -0.12 | | 0.078 | | 0.13 | | 0.054 | |
|  |  |  | |  | |  | |  | |  | |  | |  | |  | |  | |
| **Future Thinking Questionnaire** |  |  | |  | |  | |  | |  | |  | |  | |  | |  | |
|  |  |  | |  | |  | |  | |  | |  | |  | |  | |  | |
| Survey of Autobiographical Memory; Future | -0.077 | 0.26 | | -0.13 | | 0.064 | | 0.029 | | 0.67 | | 0.002 | | 0.98 | | 0.043 | | 0.53 | |
|  |  |  | |  | |  | |  | |  | |  | |  | |  | |  | |
| **Navigation Questionnaires** |  |  | |  | |  | |  | |  | |  | |  | |  | |  | |
|  |  |  | |  | |  | |  | |  | |  | |  | |  | |  | |
| Santa Barbara Sense of Direction Scale | -0.085 | 0.21 | | -0.052 | | 0.45 | | 0.12 | | 0.091 | | 0.040 | | 0.56 | | -0.089 | | 0.19 | |
| Survey of Autobiographical Memory; Spatial | -0.075 | 0.27 | | -0.092 | | 0.18 | | 0.099 | | 0.15 | | -0.008 | | 0.91 | | -0.062 | | 0.36 | |
|  |  |  | |  | |  | |  | |  | |  | |  | |  | |  | |
| **Verbal and Semantic Memory Questionnaires** |  |  | |  | |  | |  | |  | |  | |  | |  | |  | |
|  |  |  | |  | |  | |  | |  | |  | |  | |  | |  | |
| OSIVQ; Verbal | -0.14 | 0.047 | | -0.046 | | 0.50 | | 0.066 | | 0.33 | | 0.11 | | 0.12 | | -0.040 | | 0.56 | |
| Survey of Autobiographical Memory; Semantic | 0.070 | 0.31 | | -0.14 | | 0.035 | | 0.13 | | 0.049 | | 0.077 | | 0.26 | | -0.013 | | 0.84 | |
| Verbalizer | -0.24 | < 0.001 | | -0.048 | | 0.49 | | 0.042 | | 0.53 | | 0.072 | | 0.29 | | -0.011 | | 0.87 | |
|  |  |  |  | |  | |  | |  | |  | |  | |  | |  | |  |

**Table S10.** Correlation coefficients of the One Sentence Questionnaire questions with the AI internal detail sub-categories as a percentage of total utterances.

|  | **AI Events** | | **AI Place** | | **AI Time** | | **AI Perceptual** | | **AI Emotion** | |
| --- | --- | --- | --- | --- | --- | --- | --- | --- | --- | --- |
|  | **r** | **p** | **r** | **p** | **r** | **p** | **r** | **p** | **r** | **p** |
| **Imagery Questions** |  |  |  |  |  |  |  |  |  |  |
| One Sentence: Imagery Ability | -0.005 | 0.94 | -0.23 | 0.001 | 0.004 | 0.95 | -0.023 | 0.73 | 0.058 | 0.39 |
| One Sentence: Imagery Use | 0.052 | 0.44 | -0.19 | 0.004 | -0.10 | 0.13 | -0.098 | 0.15 | 0.080 | 0.24 |
| One Sentence: Imagery as a Scene | 0.002 | 0.98 | -0.19 | 0.005 | -0.039 | 0.56 | 0.006 | 0.93 | -0.030 | 0.66 |
|  |  |  |  |  |  |  |  |  |  |  |
|  |  |  |  |  |  |  |  |  |  |  |
| **Memory Questions** |  |  |  |  |  |  |  |  |  |  |
| One Sentence: Memory Ability | 0.057 | 0.40 | -0.098 | 0.15 | -0.034 | 0.62 | -0.025 | 0.72 | -0.019 | 0.78 |
| One Sentence: Memory in Imagery | 0.002 | 0.98 | -0.14 | 0.038 | 0.084 | 0.22 | 0.080 | 0.24 | -0.054 | 0.43 |
| One Sentence: Memory in Scene Imagery | 0.005 | 0.94 | -0.10 | 0.14 | 0.017 | 0.80 | 0.095 | 0.16 | -0.046 | 0.50 |
| One Sentence: Memory in Words | 0.043 | 0.53 | 0.047 | 0.49 | 0.018 | 0.79 | -0.096 | 0.16 | -0.056 | 0.41 |
|  |  |  |  |  |  |  |  |  |  |  |
| **Future Thinking Questions** |  |  |  |  |  |  |  |  |  |  |
| Future Thinking Ability | -0.086 | 0.21 | -0.11 | 0.10 | -0.012 | 0.86 | -0.026 | 0.71 | 0.085 | 0.22 |
| Future Thinking in Imagery | 0.070 | 0.30 | -0.11 | 0.10 | -0.039 | 0.57 | -0.012 | 0.87 | -0.006 | 0.94 |
| Future Thinking in Scene Imagery | -0.078 | 0.26 | -0.12 | 0.089 | -0.076 | 0.27 | 0.014 | 0.84 | 0.080 | 0.24 |
| Future Thinking in Words | -0.052 | 0.44 | 0.10 | 0.13 | 0.074 | 0.28 | -0.11 | 0.10 | -0.093 | 0.17 |
|  |  |  |  |  |  |  |  |  |  |  |
| **Navigation Questions** |  |  |  |  |  |  |  |  |  |  |
| One Sentence: Navigation Ability | -0.031 | 0.66 | -0.082 | 0.23 | 0.032 | 0.64 | -0.007 | 0.92 | -0.078 | 0.25 |
| One Sentence: Navigation in Imagery | -0.016 | 0.81 | -0.006 | 0.93 | 0.037 | 0.59 | -0.046 | 0.50 | 0.001 | 0.98 |
| One Sentence: Navigation in Scene Imagery | -0.014 | 0.83 | -0.042 | 0.54 | -0.021 | 0.76 | -0.039 | 0.57 | -0.044 | 0.52 |
| One Sentence: Navigation in Words | 0.052 | 0.45 | -0.043 | 0.52 | -0.095 | 0.16 | -0.011 | 0.87 | 0.022 | 0.75 |
|  |  | |  | |  | |  | |  | |

**Table S11.** Correlation coefficients of the memory questionnaires with the number of AI internal details for each memory time point.

|  | **Childhood** | | **Teenage** | | **“Remote”** | | **Adult** | | **Last Year** | | **“Recent”** | |
| --- | --- | --- | --- | --- | --- | --- | --- | --- | --- | --- | --- | --- |
|  | **r** | **p** | **r** | **p** | **r** | **p** | **r** | **p** | **r** | **p** | **r** | **p** |
|  |  |  |  |  |  |  |  |  |  |  |  |  |
| Memory Experience Questionnaire; Accessibility | 0.18 | 0.007 | 0.061 | 0.37 | 0.14 | 0.041 | 0.19 | 0.005 | 0.091 | 0.18 | 0.17 | 0.012 |
| Memory Experience Questionnaire; Coherence | 0.14 | 0.045 | 0.010 | 0.89 | 0.083 | 0.22 | 0.089 | 0.19 | -0.016 | 0.81 | 0.053 | 0.44 |
| Memory Experience Questionnaire; Sharing | 0.20 | 0.004 | 0.11 | 0.12 | 0.17 | 0.011 | 0.25 | < 0.001 | 0.20 | 0.003 | 0.26 | < 0.001 |
| Memory Experience Questionnaire; Vividness | 0.16 | 0.018 | 0.069 | 0.31 | 0.13 | 0.054 | 0.15 | 0.026 | 0.062 | 0.36 | 0.13 | 0.055 |
| Subjective Memory Questionnaire | 0.16 | 0.015 | 0.042 | 0.54 | 0.12 | 0.083 | 0.12 | 0.078 | -0.035 | 0.61 | 0.066 | 0.34 |
| Survey of Autobiographical Memory; Episodic | 0.14 | 0.033 | 0.079 | 0.25 | 0.13 | 0.062 | 0.13 | 0.057 | 0.010 | 0.89 | 0.092 | 0.18 |
|  |  |  |  |  |  |  |  |  |  |  |  |  |

**Table S12.** Correlation coefficients of the other group questionnaires with the number of AI internal details for each memory time point.

|  | **Childhood** | | **Teenage** | | **“Remote”** | | **Adult** | | **Last Year** | | **“Recent”** | |
| --- | --- | --- | --- | --- | --- | --- | --- | --- | --- | --- | --- | --- |
|  | **r** | **p** | **r** | **p** | **r** | **p** | **r** | **p** | **r** | **p** | **r** | **p** |
| **Imagery Questionnaires** |  |  |  |  |  |  |  |  |  |  |  |  |
|  |  |  |  |  |  |  |  |  |  |  |  |  |
| OSIVQ; Object-Scene | 0.14 | 0.041 | 0.013 | 0.85 | 0.087 | 0.20 | 0.067 | 0.33 | -0.066 | 0.33 | 0.016 | 0.82 |
| OSIVQ; Spatial | -0.072 | 0.29 | -0.035 | 0.61 | -0.061 | 0.37 | -0.15 | 0.029 | -0.11 | 0.094 | -0.15 | 0.026 |
| PSIQ; Appearance | 0.079 | 0.25 | 0.055 | 0.42 | 0.076 | 0.26 | 0.079 | 0.24 | -0.028 | 0.68 | 0.041 | 0.55 |
| Spontaneous Use of Imagery Scale | 0.11 | 0.094 | 0.028 | 0.68 | 0.081 | 0.24 | 0.031 | 0.65 | 0.011 | 0.87 | 0.026 | 0.70 |
| Visualizer | -0.036 | 0.60 | -0.069 | 0.31 | -0.059 | 0.39 | -0.14 | 0.038 | -0.13 | 0.055 | -0.15 | 0.023 |
|  |  |  |  |  |  |  |  |  |  |  |  |  |
| **Future Thinking Questionnaire** |  |  |  |  |  |  |  |  |  |  |  |  |
|  |  |  |  |  |  |  |  |  |  |  |  |  |
| Survey of Autobiographical Memory; Future | 0.070 | 0.30 | 0.058 | 0.39 | 0.073 | 0.28 | -0.018 | 0.79 | -0.098 | 0.15 | -0.056 | 0.41 |
|  |  |  |  |  |  |  |  |  |  |  |  |  |
| **Navigation Questionnaires** |  |  |  |  |  |  |  |  |  |  |  |  |
|  |  |  |  |  |  |  |  |  |  |  |  |  |
| Santa Barbara Sense of Direction Scale | 0.091 | 0.18 | 0.037 | 0.59 | 0.073 | 0.28 | -0.018 | 0.79 | -0.098 | 0.15 | -0.056 | 0.41 |
| Survey of Autobiographical Memory; Spatial | 0.055 | 0.42 | 0.026 | 0.71 | 0.046 | 0.50 | -0.037 | 0.58 | -0.097 | 0.16 | -0.069 | 0.31 |
|  |  |  |  |  |  |  |  |  |  |  |  |  |
| **Verbal and Semantic Memory Questionnaires** |  |  |  |  |  |  |  |  |  |  |  |  |
|  |  |  |  |  |  |  |  |  |  |  |  |  |
| OSIVQ; Verbal | 0.18 | 0.009 | 0.18 | 0.007 | 0.20 | 0.003 | 0.18 | 0.010 | 0.054 | 0.43 | 0.14 | 0.035 |
| Survey of Autobiographical Memory; Semantic | 0.15 | 0.025 | 0.092 | 0.18 | 0.14 | 0.041 | 0.14 | 0.034 | 0.013 | 0.85 | 0.10 | 0.13 |
| Verbalizer | 0.055 | 0.42 | 0.019 | 0.78 | 0.042 | 0.54 | 0.10 | 0.13 | 0.013 | 0.85 | 0.075 | 0.27 |
|  |  |  |  |  |  |  |  |  |  |  |  |  |

**Table S13.** Correlation coefficients of the One Sentence Questionnaire questions with the number of AI internal details for each memory time point.

|  | **Childhood** | | **Teenage** | | **“Remote”** | | **Adult** | | **Last Year** | | **“Recent”** | |
| --- | --- | --- | --- | --- | --- | --- | --- | --- | --- | --- | --- | --- |
|  | **r** | **p** | **r** | **p** | **r** | **p** | **r** | **p** | **r** | **p** | **r** | **p** |
| **Imagery Questions** |  |  |  |  |  |  |  |  |  |  |  |  |
| One Sentence: Imagery Ability | 0.14 | 0.038 | 0.065 | 0.34 | 0.12 | 0.085 | 0.072 | 0.29 | -0.037 | 0.59 | 0.032 | 0.64 |
| One Sentence: Imagery Use | 0.097 | 0.15 | 0.032 | 0.64 | 0.074 | 0.28 | 0.060 | 0.38 | -0.050 | 0.47 | 0.019 | 0.75 |
| One Sentence: Imagery as a Scene | 0.11 | 0.098 | 0.005 | 0.94 | 0.068 | 0.32 | 0.066 | 0.33 | -0.040 | 0.56 | 0.027 | 0.69 |
|  |  |  |  |  |  |  |  |  |  |  |  |  |
|  |  |  |  |  |  |  |  |  |  |  |  |  |
| **Memory Questions** |  |  |  |  |  |  |  |  |  |  |  |  |
| One Sentence: Memory Ability | 0.15 | 0.023 | 0.097 | 0.16 | 0.14 | 0.036 | 0.094 | 0.17 | 0.026 | 0.70 | 0.075 | 0.27 |
| One Sentence: Memory in Imagery | 0.18 | 0.007 | 0.074 | 0.28 | 0.15 | 0.032 | 0.082 | 0.23 | 0.068 | 0.32 | 0.086 | 0.21 |
| One Sentence: Memory in Scene Imagery | 0.11 | 0.11 | 0.073 | 0.28 | 0.10 | 0.13 | 0.066 | 0.33 | -0.061 | 0.37 | 0.018 | 0.79 |
| One Sentence: Memory in Words | -0.14 | 0.048 | 0.016 | 0.81 | -0.068 | 0.32 | -0.046 | 0.50 | -0.023 | 0.73 | -0.042 | 0.54 |
|  |  |  |  |  |  |  |  |  |  |  |  |  |
| **Future Thinking Questions** |  |  |  |  |  |  |  |  |  |  |  |  |
| Future Thinking Ability | 0.091 | 0.18 | 0.029 | 0.68 | 0.068 | 0.32 | -0.002 | 0.97 | -0.011 | 0.88 | -0.006 | 0.93 |
| Future Thinking in Imagery | 0.11 | 0.12 | 0.013 | 0.85 | 0.069 | 0.32 | 0.053 | 0.44 | 0.019 | 0.78 | 0.044 | 0.52 |
| Future Thinking in Scene Imagery | 0.062 | 0.36 | 0.037 | 0.59 | 0.056 | 0.41 | 0.007 | 0.92 | -0.045 | 0.51 | -0.016 | 0.82 |
| Future Thinking in Words | -0.038 | 0.58 | 0.048 | 0.48 | 0.005 | 0.94 | 0.025 | 0.71 | -0.031 | 0.65 | 0.003 | 0.96 |
|  |  |  |  |  |  |  |  |  |  |  |  |  |
| **Navigation Questions** |  |  |  |  |  |  |  |  |  |  |  |  |
| One Sentence: Navigation Ability | 0.13 | 0.060 | 0.062 | 0.36 | 0.11 | 0.11 | -0.048 | 0.48 | -0.034 | 0.62 | -0.048 | 0.48 |
| One Sentence: Navigation in Imagery | 0.078 | 0.25 | 0.055 | 0.42 | 0.076 | 0.27 | 0.018 | 0.79 | -0.040 | 0.55 | -0.006 | 0.93 |
| One Sentence: Navigation in Scene Imagery | 0.062 | 0.36 | 0.11 | 0.10 | 0.098 | 0.15 | 0.082 | 0.23 | 0.031 | 0.65 | 0.070 | 0.31 |
| One Sentence: Navigation in Words | -0.002 | 0.98 | 0.056 | 0.41 | 0.030 | 0.66 | 0.10 | 0.14 | 0.091 | 0.18 | 0.11 | 0.11 |
|  |  |  |  | |  | |  | |  | |  | |

**Table S14.** Correlation coefficients of the memory questionnaires with the total number of AI internal details for each internal detail sub-category.

|  | **AI Internal Details** | | **AI Events** | | **AI Place** | | **AI Time** | | **AI Perceptual** | | **AI Emotion** | |
| --- | --- | --- | --- | --- | --- | --- | --- | --- | --- | --- | --- | --- |
|  | **r** | **p** | **r** | **p** | **r** | **p** | **r** | **p** | **r** | **p** | **r** | **p** |
|  |  |  |  |  |  |  |  |  |  |  |  |  |
| Memory Experience Questionnaire; Accessibility | 0.18 | 0.009 | 0.17 | 0.015 | 0.049 | 0.47 | 0.21 | 0.002 | 0.099 | 0.15 | 0.095 | 0.17 |
| Memory Experience Questionnaire; Coherence | 0.071 | 0.30 | 0.057 | 0.40 | 0.055 | 0.42 | 0.23 | 0.001 | 0.046 | 0.50 | -0.025 | 0.71 |
| Memory Experience Questionnaire; Sharing | 0.26 | < 0.001 | 0.25 | < 0.001 | 0.093 | 0.17 | 0.19 | 0.006 | 0.13 | 0.057 | 0.18 | 0.009 |
| Memory Experience Questionnaire; Vividness | 0.15 | 0.033 | 0.15 | 0.033 | 0.009 | 0.90 | 0.19 | 0.005 | 0.041 | 0.55 | 0.14 | 0.047 |
| Subjective Memory Questionnaire | 0.093 | 0.17 | 0.051 | 0.46 | 0.055 | 0.42 | 0.17 | 0.015 | 0.096 | 0.16 | 0.022 | 0.75 |
| Survey of Autobiographical Memory; Episodic | 0.12 | 0.088 | 0.12 | 0.087 | -0.01 | 0.89 | 0.22 | 0.001 | 0.045 | 0.51 | 0.063 | 0.35 |
|  |  |  |  |  |  |  |  |  |  |  |  |  |

**Table S15.** Correlation coefficients of the other group questionnaires with the total number of AI internal details for each internal detail sub-category.

|  | **AI Internal Details** | | **AI Events** | | **AI Place** | | | | **AI Time** | | | | **AI Perceptual** | | | | **AI Emotion** | | | |  |
| --- | --- | --- | --- | --- | --- | --- | --- | --- | --- | --- | --- | --- | --- | --- | --- | --- | --- | --- | --- | --- | --- |
|  | **r** | **p** | **r** | **p** | | **r** | | **p** | | **r** | | **p** | | **r** | | **p** | | **r** | | **p** | |
| **Imagery Questionnaires** |  |  |  |  | |  | |  | |  | |  | |  | |  | |  | |  | |
|  |  |  |  |  | |  | |  | |  | |  | |  | |  | |  | |  | |
| OSIVQ; Object-Scene | 0.045 | 0.51 | 0.062 | 0.36 | | -0.025 | | 0.72 | | 0.16 | | 0.019 | | -0.029 | | 0.67 | | 0.050 | | 0.47 | |
| OSIVQ; Spatial | -0.13 | 0.051 | -0.11 | 0.12 | | -0.15 | | 0.032 | | -0.053 | | 0.44 | | -0.14 | | 0.035 | | 0.007 | | 0.91 | |
| PSIQ; Appearance | 0.060 | 0.38 | 0.062 | 0.37 | | -0.084 | | 0.22 | | 0.16 | | 0.021 | | 0.039 | | 0.57 | | 0.011 | | 0.87 | |
| Spontaneous Use of Imagery Scale | 0.051 | 0.46 | 0.057 | 0.40 | | -0.020 | | 0.77 | | 0.086 | | 0.21 | | -0.010 | | 0.89 | | 0.077 | | 0.26 | |
| Visualizer | -0.13 | 0.049 | -0.11 | 0.10 | | -0.12 | | 0.068 | | -0.062 | | 0.36 | | -0.15 | | 0.024 | | 0.031 | | 0.65 | |
|  |  |  |  |  | |  | |  | |  | |  | |  | |  | |  | |  | |
| **Future Thinking Questionnaire** |  |  |  |  | |  | |  | |  | |  | |  | |  | |  | |  | |
|  |  |  |  |  | |  | |  | |  | |  | |  | |  | |  | |  | |
| Survey of Autobiographical Memory; Future | 0.029 | 0.67 | 0.011 | 0.87 | | -0.055 | | 0.42 | | 0.084 | | 0.22 | | 0.018 | | 0.79 | | 0.051 | | 0.45 | |
|  |  |  |  |  | |  | |  | |  | |  | |  | |  | |  | |  | |
| **Navigation Questionnaires** |  |  |  |  | |  | |  | |  | |  | |  | |  | |  | |  | |
|  |  |  |  |  | |  | |  | |  | |  | |  | |  | |  | |  | |
| Santa Barbara Sense of Direction Scale | -0.012 | 0.86 | -0.034 | 0.62 | | -0.054 | | 0.43 | | 0.13 | | 0.053 | | 0.038 | | 0.57 | | -0.076 | | 0.26 | |
| Survey of Autobiographical Memory; Spatial | -0.031 | 0.65 | -0.036 | 0.60 | | -0.086 | | 0.21 | | 0.14 | | 0.036 | | -0.002 | | 0.98 | | -0.075 | | 0.27 | |
|  |  |  |  |  | |  | |  | |  | |  | |  | |  | |  | |  | |
| **Verbal and Semantic Memory Questionnaires** |  |  |  |  | |  | |  | |  | |  | |  | |  | |  | |  | |
|  |  |  |  |  | |  | |  | |  | |  | |  | |  | |  | |  | |
| OSIVQ; Verbal | 0.18 | 0.007 | 0.089 | 0.19 | | 0.12 | | 0.079 | | 0.18 | | 0.007 | | 0.20 | | 0.003 | | 0.093 | | 0.17 | |
| Survey of Autobiographical Memory; Semantic | 0.13 | 0.058 | 0.12 | 0.089 | | -0.088 | | 0.20 | | 0.19 | | 0.004 | | 0.11 | | 0.11 | | 0.041 | | 0.55 | |
| Verbalizer | 0.071 | 0.30 | -0.032 | 0.64 | | 0.066 | | 0.34 | | 0.14 | | 0.047 | | 0.12 | | 0.089 | | 0.086 | | 0.21 | |
|  |  |  |  |  |  | |  | |  | |  | |  | |  | |  | |  | |  |

**Table S16.** Correlation coefficients of the One Sentence Questionnaire questions with the total number of AI internal details for each internal detail sub-category.

|  | **AI Internal Details** | | **AI Events** | | **AI Place** | | **AI Time** | | **AI Perceptual** | | **AI Emotion** | |
| --- | --- | --- | --- | --- | --- | --- | --- | --- | --- | --- | --- | --- |
|  | **r** | **p** | **r** | **p** | **r** | **p** | **r** | **p** | **r** | **p** | **r** | **p** |
| **Imagery Questions** |  |  |  |  |  |  |  |  |  |  |  |  |
| One Sentence: Imagery Ability | 0.069 | 0.31 | 0.066 | 0.33 | -0.099 | 0.15 | 0.081 | 0.24 | 0.021 | 0.76 | 0.11 | 0.10 |
| One Sentence: Imagery Use | 0.042 | 0.53 | 0.098 | 0.15 | -0.10 | 0.13 | -0.034 | 0.62 | -0.045 | 0.51 | 0.091 | 0.18 |
| One Sentence: Imagery as a Scene | 0.046 | 0.50 | 0.072 | 0.29 | -0.074 | 0.28 | 0.032 | 0.64 | 0.021 | 0.76 | 0.009 | 0.89 |
|  |  |  |  |  |  |  |  |  |  |  |  |  |
|  |  |  |  |  |  |  |  |  |  |  |  |  |
| **Memory Questions** |  |  |  |  |  |  |  |  |  |  |  |  |
| One Sentence: Memory Ability | 0.11 | 0.11 | 0.13 | 0.054 | 0.044 | 0.52 | 0.070 | 0.31 | 0.052 | 0.45 | 0.032 | 0.64 |
| One Sentence: Memory in Imagery | 0.12 | 0.081 | 0.083 | 0.22 | 0.010 | 0.88 | 0.18 | 0.009 | 0.12 | 0.085 | 0.029 | 0.67 |
| One Sentence: Memory in Scene Imagery | 0.053 | 0.44 | 0.034 | 0.62 | -0.038 | 0.58 | 0.068 | 0.32 | 0.089 | 0.19 | -0.026 | 0.70 |
| One Sentence: Memory in Words | -0.057 | 0.41 | -0.013 | 0.85 | 0.008 | 0.91 | -0.005 | 0.94 | -0.090 | 0.19 | -0.051 | 0.46 |
|  |  |  |  |  |  |  |  |  |  |  |  |  |
| **Future Thinking Questions** |  |  |  |  |  |  |  |  |  |  |  |  |
| Future Thinking Ability | 0.022 | 0.75 | 0.003 | 0.96 | -0.061 | 0.37 | 0.035 | 0.61 | 0.006 | 0.93 | 0.086 | 0.21 |
| Future Thinking in Imagery | 0.059 | 0.39 | 0.10 | 0.14 | -0.023 | 0.74 | 0.012 | 0.86 | 0.005 | 0.94 | 0.016 | 0.81 |
| Future Thinking in Scene Imagery | 0.011 | 0.88 | -0.006 | 0.93 | -0.061 | 0.37 | -0.035 | 0.61 | 0.004 | 0.95 | 0.089 | 0.19 |
| Future Thinking in Words | 0.004 | 0.95 | 0.016 | 0.82 | 0.16 | 0.023 | 0.085 | 0.21 | -0.048 | 0.49 | -0.023 | 0.74 |
|  |  |  |  |  |  |  |  |  |  |  |  |  |
| **Navigation Questions** |  |  |  |  |  |  |  |  |  |  |  |  |
| One Sentence: Navigation Ability | 0.008 | 0.91 | 0.011 | 0.88 | -0.013 | 0.85 | 0.076 | 0.27 | 0.013 | 0.85 | -0.040 | 0.56 |
| One Sentence: Navigation in Imagery | 0.026 | 0.71 | 0.019 | 0.78 | 0.071 | 0.30 | 0.079 | 0.25 | -0.015 | 0.83 | 0.035 | 0.61 |
| One Sentence: Navigation in Scene Imagery | 0.089 | 0.19 | 0.10 | 0.14 | 0.096 | 0.16 | 0.064 | 0.35 | 0.028 | 0.68 | 0.037 | 0.59 |
| One Sentence: Navigation in Words | 0.090 | 0.19 | 0.12 | 0.070 | 0.023 | 0.74 | -0.078 | 0.25 | 0.022 | 0.75 | 0.087 | 0.20 |
|  |  |  |  | |  | |  | |  | |  | |

**One Sentence Questionnaire**

1. Please rate your ability to construct a mental image (circle one)

Very High High Fairly High Average Fairly Low Low Very low

1. In everyday life, how much do you think in images (e.g. thinking in pictures in your mind)

Not at all 1 2 3 4 5 6 7 All the time

1. If you think in images, to what extent does this involve spatially coherent scenes (e.g. scenes that you could step into or operate within) compared to single objects?

Single objects 1 2 3 4 5 6 7 Coherent scene

1. Please rate your ability to remember your personal past (circle one)

Very High High Fairly High Average Fairly Low Low Very low

1. When recalling the past, to what extent do you think in images

Not at all 1 2 3 4 5 6 7 All the time

1. If you think in images, when recalling the past to what extent do you evoke spatially coherent scenes in your mind's eye, compared to imagining single objects

Single Objects 1 2 3 4 5 6 7 Coherent Scene

1. When recalling the past, how much do you think verbally (e.g. thinking in words and sentences)

Not at all 1 2 3 4 5 6 7 All the time

1. Please rate your ability to imagine future events (circle one)

Very High High Fairly High Average Fairly Low Low Very low

1. When imagining the future, to what extent do you think in images

Not at all 1 2 3 4 5 6 7 All the time

1. If you think in images, when imagining the future to what extent do you evoke spatially coherent scenes in your mind's eye, compared to imagining single objects

Single Objects 1 2 3 4 5 6 7 Coherent Scene

1. When imagining the future, how much do you think verbally (e.g. thinking in words and sentences)

Not at all 1 2 3 4 5 6 7 All the time

1. Please rate your navigational ability (circle one)

Very High High Fairly High Average Fairly Low Low Very low

1. When you navigate, to what extent do you think in images

Not at all 1 2 3 4 5 6 7 All the time

1. If you think in images, when navigating to what extent do you evoke spatially coherent scenes in your mind's eye, compared to imagining single objects

Single Objects 1 2 3 4 5 6 7 Coherent Scene

1. When navigating, how much do you think verbally (e.g. thinking in words and sentences)

Not at all 1 2 3 4 5 6 7 All the time

**References**

Hassabis, D., Kumaran, D., Vann, S. D., & Maguire, E. A. (2007). Patients with hippocampal amnesia cannot imagine new experiences. *Proceedings of the National Academy of Sciences, 104*(5), 1726-1731. doi: <http://dx.doi.org/10.1073/pnas.0610561104>

Levine, B., Svoboda, E., Hay, J. F., Winocur, G., & Moscovitch, M. (2002). Aging and autobiographical memory: Dissociating episodic from semantic retrieval. *Psychology and Aging, 17*(4), 677-689. doi: <http://dx.doi.org/10.1037/0882-7974.17.4.677>

Woollett, K., & Maguire, E. A. (2010). The effect of navigational expertise on wayfinding in new environments. *Journal of Environmental Psychology, 30*(4), 565-573. doi: <http://dx.doi.org/10.1016/j.jenvp.2010.03.003>
